# Limbic encephalitis associated with AMPA receptor and CRMP5 antibodies: A case report and literature review

**DOI:** 10.1002/brb3.1528

**Published:** 2020-01-28

**Authors:** Yujuan Jia, Jie Wang, Lanping Xue, Yuli Hou

**Affiliations:** ^1^ Department of Neurology First Hospital of Shanxi Medical University Taiyuan China; ^2^ Department of Neurology Shanxi Bethune Hospital Taiyuan China

**Keywords:** AMPAR, antibodies, CRMP5, limbic encephalitis, malignant tumor

## Abstract

**Aims:**

AMPA receptor (AMPAR) and CRMP5 antibodies are relatively uncommon in limbic encephalitis, and patients with both antibodies are rare. We recently treated such a patient, but the patient died after active treatment. To further understand this disease, we conducted a case report and literature review.

**Discussions:**

To date, five encephalitis patients, including our patient, have been found to be positive for AMPAR and CRMP5 antibodies. The male‐to‐female ratio of the reported cases is 4:1, and the age range is 26 and 62 years old. All five patients presented with various neuropsychiatric symptoms, including insomnia, abnormal behavior, seizures, extrapyramidal symptoms, and autonomic dysfunction. Four patients had tumors (three invasive thymomas and one suspected lymphoma), and three cases died within a short period of time. No tumor was detected in one of the patients during the follow‐up period; however, after active treatment, the outcome was poor, and the patient developed cachexia. One patient had good response to immunotherapy and tumor therapy and successfully returned to work.

**Conclusions:**

The prognosis of encephalitis associated with AMPAR and CRMP5 antibodies is worse than that of the encephalitis associated with AMPAR antibodies alone. The most likely cause is that this encephalitis is more likely to be accompanied by malignant tumors, leading to a poor prognosis. In addition, it may also be due to some synergistic mechanisms between the two antibodies. Further studies aimed at the prognosis of this type of encephalitis are warranted.

## Graphical abstract

The prognosis of encephalitis associated with AMPA receptor (AMPAR) and CRMP5 antibodies is worse than that of the encephalitis associated with AMPAR antibodies alone. The most likely cause is that this encephalitis is more likely to be accompanied by malignant tumors, leading to a poor prognosis. Further studies aimed at the prognosis of this type of encephalitis are warranted.

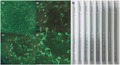

## 1 INTRODUCTION

Limbic encephalitis (LE) is an inflammatory disease arising from selective involvement of medial temporal lobe, orbitofrontal cortex, and amygdala. Its main clinical manifestations include memory loss, behavioral abnormalities, epilepsy, and, in some cases, dementia (Dalmau & Vincent, 2017). Most patients with LE present electroencephalogram (EEG) or magnetic resonance imaging (MRI) abnormalities in the limbic system of the brain and cerebrospinal fluid (CSF) inflammatory findings, and antineuronal antibodies are often present. These antibodies target two broad categories of antigens and are correspondingly divided into two major types. One type targets intracellular antigens, including Hu (or ANNA1), Ri (ANNA2), Yo (PCA1), CV2/CRMP5, Ma2, and amphiphysin. The other type targets cell membrane antigens, including N‐methyl‐D‐aspartate receptor (NMDAR), the voltage‐gated potassium channel (VGKC) receptor, GABA type B receptors (GABABRs), the alpha‐amino‐3‐hydroxy‐5‐methyl‐4‐isoxazolepropionic acid receptor (AMPAR), and glycine receptors (GlyRs; Seluk et al., 2019; Tuzun & Dalmau, 2007). The vast majority of patients have only one type of antibody, and the presence of two or more antibodies is rare. Here, we report a case of LE positive for both AMPAR and CRMP5 antibodies and review the related literature to explain the possible pathogenesis and poor prognosis of this condition.

## 2 CASE REPORT

A 26‐year‐old previously healthy man presented to an outside hospital with a history of insomnia, confusion, involuntary movements, psychiatric symptoms, and urinary retention for 3 weeks. An extensive work‐up completed at the outside hospital indicated hyponatremia (sodium concentration: 110.8 mmol/L) and atrial tachycardia (HR: 124 b/min). Due to progressive aggravation of the disease, he was referred to our hospital (18 December 2018). Upon admission, he presented consciousness disturbance and had difficulty following commands. A physical neurological examination revealed that he exhibited visible involuntary movement of the limbs and had high muscle tension in the limbs, corresponding hyperactive deep tendon reflexes and bilateral Babinski response.

**Figure 1:**
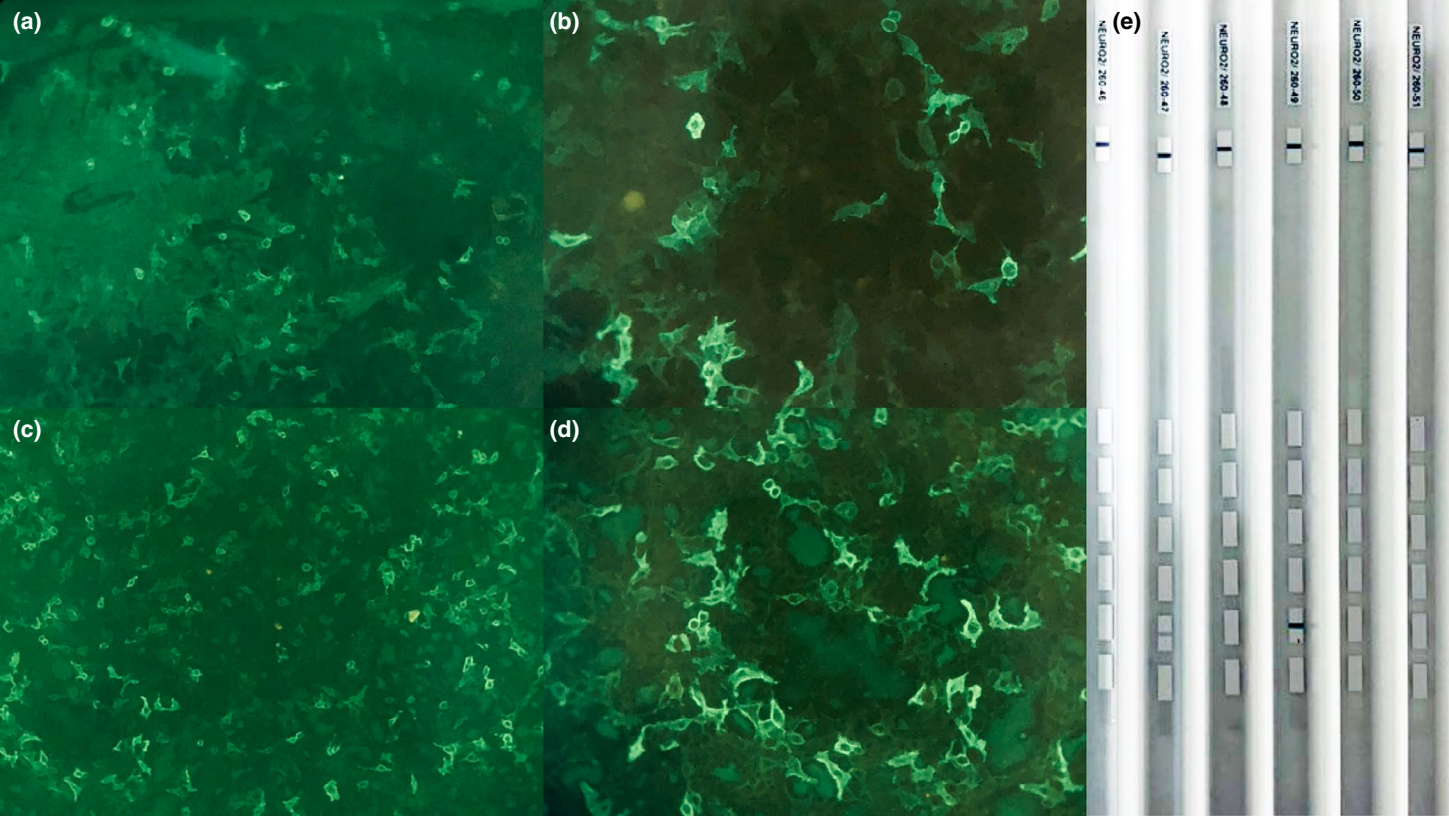
HEK293 cells expressing AMPA receptor (AMPAR GluR2) in the serum (a and b) and CSF (c and d). The titre of antibodies in the serum and CSF was measured as 1:32 (a‐d) (original magnification ×10 or 20). The serum (the third column on the right) and CSF (the second column on the left) were positive for CRMP5 antibodies by western blot (e)

**Figure 2:**
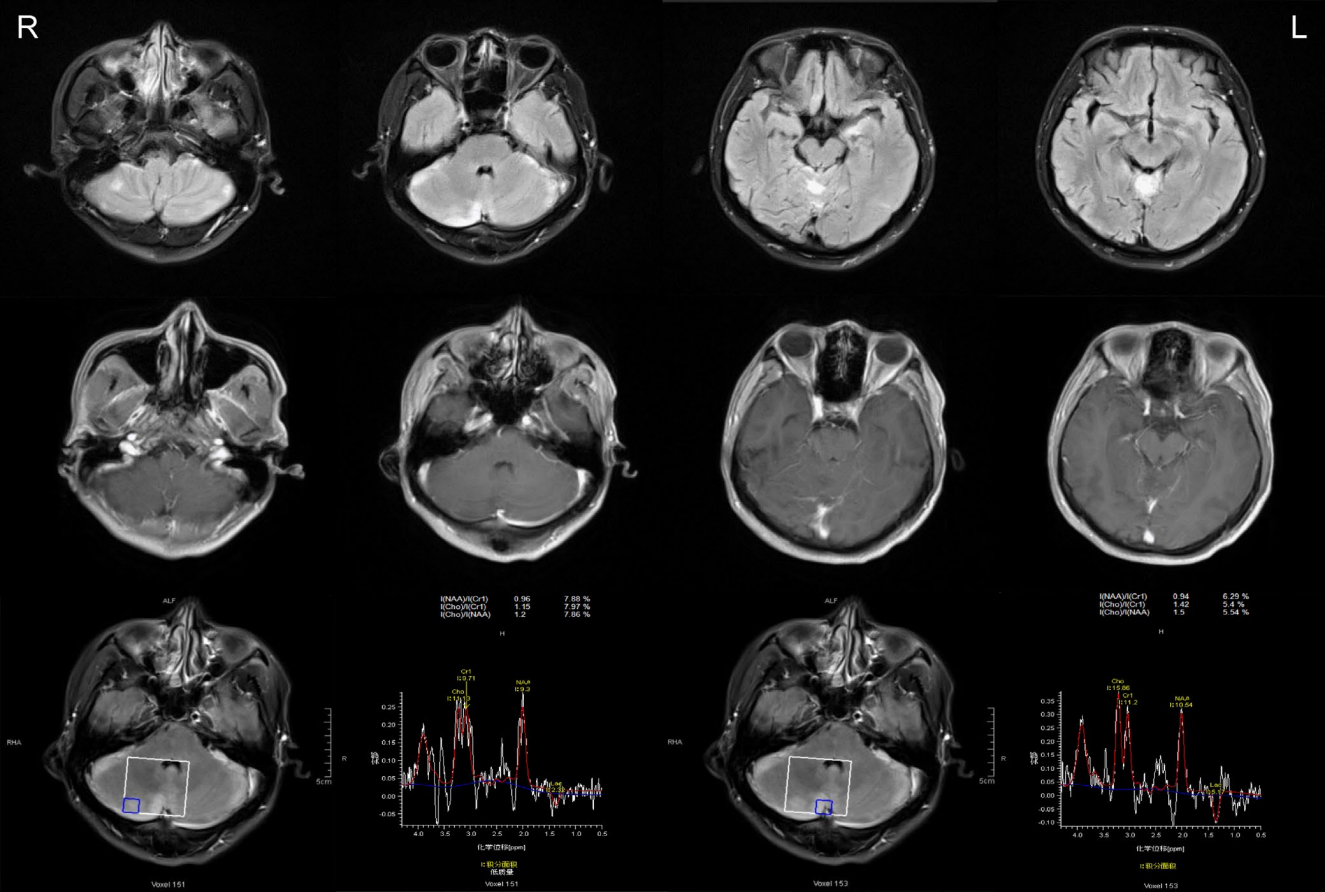
Two brain magnetic resonance imaging (MRI) scans obtained 3 weeks after symptom onset showed increased fluid‐attenuated inversion recovery (FLAIR) signal abnormalities involving the bilateral cerebellar hemispheres, cerebellar vermis, left hippocampus, basal ganglia region and bilateral frontal parietal cortex (the first row); however, no obvious enhancement was observed (the second row). Spectra for voxels in the cerebellar hemispheres showed a markedly reduced N‐acetyl aspartate (NAA) peak and NAA/choline (Cho) ratio (the third row)

**Figure 3:**
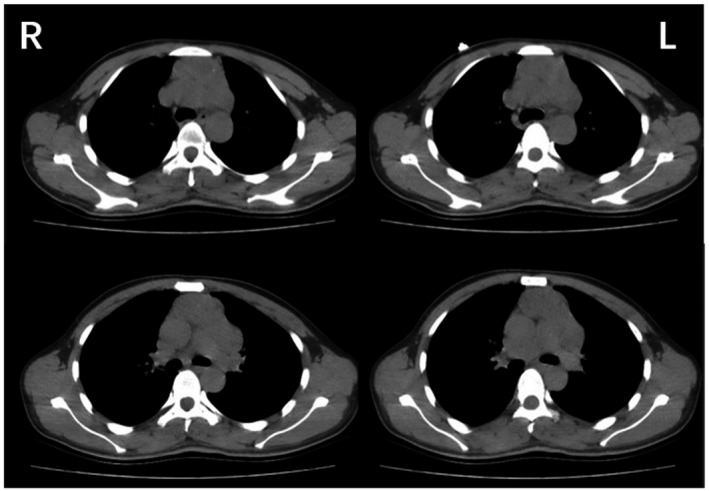
Chest computed tomography (CT) scan showing a mass in the anterosuperior mediastinum. Most of the lesions, which were wavy and fused together, were located in the anterior superior mediastinum

Cerebrospinal fluid analysis revealed 11 leukocytes per µl mostly lymphocytes (87%), none red blood cells, and normal biochemistry. Paraneoplastic antibodies in serum and CSF were all negative, including anti‐Hu, anti‐Yo, anti‐Ri, antiMa2, and antiamphiphysin; besides, anti‐CV2/CRMP5 antibodies were positive (Figure 1e). Simultaneously, neuropil antibodies (anti‐NMDAR, anti‐AMPAR1, anti‐AMPAR2, anti‐GABABR, anti‐LGI1, anti‐CASPR2, and anti‐GAD65) in serum and CSF were also tested. Among these antibodies, AMPAR2 antibodies were detected both in the serum and CSF (Figure 1a–d). When arrived at our hospital, the patient underwent a brain MRI. The brain MRI showed increased T2/fluid‐attenuated inversion recovery (FLAIR)/diffusion‐weighted imaging (DWI) signal abnormalities involving the bilateral cerebellar hemispheres, cerebellar vermis, left hippocampus, basal ganglia region, and bilateral frontoparietal cortex; however, no obvious enhancement was observed. Spectra for voxels in the cerebellar hemispheres showed a markedly reduced N‐acetyl aspartate (NAA) peak and NAA/choline (Cho) ratio (Figure 2). A chest computed tomography (CT) scan showed a mass in the anterosuperior mediastinum (Figure 3). Because most of the lesions were wavy and fused together and located in the anterior superior mediastinum, the mass was suspected to be lymphoma.

On the day of admission, both glucocorticoid (1 g/day) and intravenous immunoglobulin (IVIg; 0.4 mg/kg) therapy were administered. However, the patient's condition continued to deteriorate: His heart rate continued to increase (120–140 b/min), his blood pressure was slightly elevated, at 172/90 (130–172/90–120 mm Hg), his temperature continuously increased (constantly maintained at 41°C), his hyponatremia continued (116–122 mmol/L), and he presented progressive consciousness disruption and involuntary limb movement. On the second day after admission, the patient suffered from Cheyne–Strokes breathing and had difficulty breathing, and his blood oxygen saturation continued to decline. Tracheal intubation was performed, and ventilator‐assisted breathing was initiated. On the same day, the patient experienced a tonic–clonic seizure. The patient's condition deteriorated rapidly, and respiratory failure and arrhythmia soon appeared. Early on the morning of the 4th day after admission (23 December 2018), the patient suffered from circulatory and respiratory failure and died.

## 3 DISCUSSION

In recent years, an increasing number of autoimmune antibodies associated with LE have been identified, and some of these antibodies have led to a variety of paraneoplastic neurological disorders (PND). Some antibodies are clearly associated with specific neurological disorders; for example, anti‐Tr and anti‐Yo antibodies are clearly associated with cerebellar ataxia (Grativvol, Cavalcante, Castro, Nitrini, & Simabukuro, 2018; Honnorat & Cartalat, 2009). However, because patients with two or more such antibodies are very rare, the characteristics of these patients remain unclear. Here, we report a case of LE positive for both AMPAR and CRMP5 antibodies and review the related literature to explain the possible pathogenesis and poor prognosis of the disease. To date, LE associated with AMPAR and CRMP5 antibodies has been reported in only five cases, including ours (Table 1). The first case was a 53‐year‐old female patient who was diagnosed with malignant thymoma and treated with chemotherapy and radiation (Hoftberger et al., 2015). She presented with LE several months later and died suddenly of pulmonary embolism. The second case, a 62‐year‐old male patient, presented with LE, but no tumor was identified. He received steroids and rituximab but developed cachexia (Hoftberger et al., 2015). The third case was a 40‐year‐old male with a suspected malignant thymoma who was treated with IVIg but showed no substantial clinical improvement. Finally, the patient's family refused further treatment, and the patient died 2 months later (Yang et al., 2016). The fourth case was a 44‐year‐old man with thymoma who was treated with thymectomy, methylprednisolone, IVIg, and rituximab. Finally, he successfully returned to work as a business manager (Laurido‐Soto et al., 2019). In contrast to the other four cases, our patient was a 26‐year‐old male who was diagnosed with suspected lymphoma. He received steroids and IVIg treatment, but he died shortly after the initiation of the first cycle of treatment. Thus, it can be seen this kind of encephalitis can affect different people in different ways. LE can invade the cerebral cortex, limbic system, basal ganglia, and cerebellum and manifest as various neuropsychiatric symptoms, such as insomnia, confusion, psychosis, abnormal behavior, generalized seizure, autonomic dysfunction, ataxia, involuntary movements, and optic neuropathy. LE is often associated with malignant thymoma and may also be associated with other malignancies, such as lymphoma. Due to the limited case reports, the statistical results showed that this type of encephalitis had a mortality rate of nearly 60%. Additionally, tumor was identified in 80% cases. Because AMPAR and CRMP5 antibodies are relatively rare in LE, the characteristics of the single‐antibody‐positive (AMPAR or CRMP5 antibodies) encephalitis are not entirely clear. Osvaldo Laurido‐Soto summarized the characteristics of anti‐AMPAR encephalitis in 2018 through literature review. His results showed that the mortality rate of anti‐AMPAR encephalitis was 16% (9/55). While tumor was identified in 67% cases (Laurido‐Soto et al., 2019). Unfortunately, there are no similar studies on anti‐CRMP5 encephalitis. Compared with anti‐AMPAR encephalitis, encephalitis associated with AMPAR and CRMP5 antibodies has a higher mortality rate. The mechanism is complex. It is suspected that coexisting AMPAR and CRMP5 antibodies may be more likely to be associated with malignancies, resulting in adverse prognosis.

**Table 1:** Characteristics of patients with limbic encephalitis associated with AMPA receptor and CRMP5 antibodies

No.	Age, year/sex	Clinical presentation	Initial MRI	ECG	CSF	Tumor	Treatment	Time‐to‐treatment (week)	Follow‐up (week), outcome
1	53/F	Confusion, bradypsychia, status epilepticus, autonomic dysfunction	Increased signal in medial temporal lobes, frontobasal and caudate regions	NA	164 WBC, 92 mg/dl protein	Malignant thymoma	Tumor resection, chemotherapy, radiotherapy, steroids, IVIg	1	5, Patient died
2	62/M	Short‐term memory loss, confusion, abnormal behavior, psychosis, optic neuropathy, insomnia, ataxia	Hyperintensities in basal ganglia	Focal activity	33 WBC, 173 mg/dl protein	NA	Steroids, rituximab	21	30, Patient developed cachexia
3	40/M	Dementia, confusion, generalized seizure, numbness and weakness in left limbs, involuntary movements, autonomic dysfunction	Hyperintensity in the right temporal and parietal cortex	Unremarkable	52 WBC, 63.2 mg/dl protein	Suspected malignant thymoma	IVIg	2	11, Patient died
4	44/M	Disorientation, forgetfulness, labile mood, hallucinations and dystonia	Hyperintensity in bilateral hippocampal	Generalized slowing	47 mg/dl protein	thymoma	Tumor resection, steroids, IVIg, rituximab	immediately	60, successfully returned to work
5	26/M	Insomnia, bradypsychia, psychosis, confusion, generalized seizure, involuntary movements, ataxia, autonomic dysfunction, hyponatremia	Increased signal in bilateral cerebellar hemispheres, cerebellar vermis, left hippocampus, basal ganglia and bilateral frontoparietal cortex, no obvious enhancement	Unremarkable	52 WBC, 37.7 mg/dl protein	Suspected lymphoma	Steroids, IVIg	3	3, Patient died

Abbreviations: CSF, cerebrospinal fluid; MRI, magnetic resonance imaging.

Limbic encephalitis cases positive for AMPAR antibodies were first described in a 2009 report of 10 patients (Lai et al., 2009). AMPAR is an ionic glutamate receptor that is highly conserved in mammals and mediates fast excitatory neurotransmission in the brain (Shepherd & Huganir, 2007). The highest level of AMPAR is the hippocampal synaptic CA3‐CA1 region, followed by the subiculum, cerebellum, caudate‐putamen, and cerebral cortex (Laurido‐Soto et al., 2019). A previous study showed that binding of AMPAR antibodies to specific receptors on the synapse caused in an inward movement of the AMPARs on the synapse, resulting in a decrease in AMPARs in the synapse. In contrast, AMPAR antibodies did not affect the number or activity of excitatory synapses or NMDAR clusters, leading to decreased inhibition of synaptic homeostasis and increased intrinsic excitability and resulting in corresponding clinical symptoms (Bataller & Escrig, 2010; Gleichman, Panzer, Baumann, Dalmau, & Lynch, 2014; Xiaoyu et al., 2015). However, the reversibility of AMPARs also explains the good response to immunotherapy, such as corticosteroids, intravenous immunoglobulin, or plasma exchange, observed in AMPAR encephalitis. Additionally, studies have shown that patients with tumor and additional paraneoplastic autoimmunity have very high mortality (6/7 patients vs. 2/17 with cancer and isolated AMPAR antibodies; Panzer & Dale, 2015). Similarly, it has been suggested that the coexistence of onconeural antibodies predicted a poor outcome (Höftberger et al., 2015). The explanation for the poor prognoses observed in affected patients was an accompanying additional immune response, especially those involving cytotoxic T‐cell mechanisms (Bernal et al., 2002; Josep & Rosenfeld, 2008).

Limbic encephalitis positive for CRMP5 antibodies was first described in 1993 in a study that described a patient diagnosed with paraneoplastic encephalomyelitis that presented as uveitis, cerebellar ataxia, peripheral neuropathy, and an undifferentiated carcinoma (Antoine et al., 1993). Anti‐CRMP5 is a member of the collapsin response mediator family (Dericioglu, Gocmen, & Tan, 2018). CRMP5 is highly expressed during brain development and regulates the polarity of neurons by inhibiting the growth of dendrites, thereby exerting its physiological effects. However, in adulthood, the expression of CRMP5 is restricted to areas of the brain that retain neurogenesis, and thus its number becomes significantly reduced in the adult brain. CRMP5 is an intracellular autoimmune antibody expressed mainly in the cortex, hippocampus, and cerebellum (Brot et al., 2014). Studies have shown that CRMP5 is an autoimmune antibody associated with paraneoplastic neurological syndrome (PNS) and often associated with small cell lung cancer or thymoma (Brot et al., 2013; Rajesh & Bernhard, 2013). Honnorat reported that the frequency of cancer with CRMP‐5‐IgG alone was 86% (Horta et al., 2014). PNS associated with anti‐CRMP5 is generally characterized by optic neuritis, posterior uveitis, cerebellar ataxia, and chorea. It was also reported that patients with anti‐CRMP5 antibodies had good Rankin scores and long median survival times (Honnorat & Cartalat, 2009); however, the specific immune mechanism underlying this relationship is not clear. Studies have shown that PNDs occur due to an immune mechanism involving cytotoxic T cells and antibodies against target neuronal proteins that are usually expressed by an underlying tumor (Melzer & Wiendl, 2013). Previous studies have shown that CD8+ T cells present with a predominantly perivascular localization in CRMP5‐IgG positive encephalitis (Gold, Pul, Bach, Stangel, & Dodel, 2012). Some indirect evidence, including pathology studies, have suggested that extensive T‐cell infiltration of the central nervous system (CNS) occurs in these patients, supporting the hypothesis that cytotoxic T‐cell mechanisms lead to irreversible neuronal damage (Gold et al., 2012; Melzer & Wiendl, 2013; Tomotaka & Shoji, 2010).

Limbic encephalitis positive for either AMPAR or CRMP5 antibodies is a rare condition. Patients with both AMPAR and CRMP5 antibodies are extremely rare; and no detailed data are available on the patient demographics, clinical phenotypes, or treatment efficacy of this population. However, it is suggested that the prognosis of encephalitis associated with AMPAR and CRMP5 antibodies is worse than that of single‐antibody‐positive (AMPAR or CRMP5 antibodies) encephalitis. The most likely cause is that this encephalitis is more likely to be accompanied by malignant tumors, leading to a poor prognosis. In addition, it may also be due to some synergistic mechanisms between the two antibodies, resulting in poor prognosis. However, as long‐term outcomes were inversely related to time‐to‐treatment, it was also possible that delayed treatment and inadequate medication had led to the poor prognosis. Last but not least, due to the limited number of reported cases, the effect of publication bias cannot be ruled out. Thus, more clinical cases need to be reported. Further studies investigating the relationship between AMPAR and CRMP5 antibodies are warranted.

## CONFLICT OF INTEREST

None declared.

## Data Availability

The data that support the findings of this study are openly available in references number 14, 18, and 24.

## References

[brb31528-bib-0001] Antoine, J. C. , Honnorat, J. , Vocanson, C. , Koenig, F. , Aguera, M. , Belin, M. F. , & Michel, D. (1993). Posterior uveitis, paraneoplastic encephalomyelitis and auto‐antibodies reacting with developmental protein of brain and retina. Journal of the Neurological Sciences, 117(1–2), 215–223. 10.1016/0022-510X(93)90176-Y 8410058

[brb31528-bib-0002] Bataller, L. , & Escrig, G. R. (2010). Reversible paraneoplastic limbic encephalitis associated with antibodies to the AMPA receptor. Neurology, 74(3), 265–267. 10.1212/WNL.0b013e3181cb3e52 20083804 PMC2809037

[brb31528-bib-0003] Bernal, F. , Graus, F. , Pifarré, À. , Saiz, A. , Benyahia, B. , & Ribalta, T. (2002). Immunohistochemical analysis of anti‐Hu‐associated paraneoplastic encephalomyelitis. Acta Neuropathologica, 103(5), 509–515. 10.1007/s00401-001-0498-0 11935268

[brb31528-bib-0004] Brot, S. , Auger, C. , Bentata, R. , Rogemond, V. , Ménigoz, S. , Chounlamountri, N. , … Moradi‐Améli, M. (2014). Collapsin response mediator protein 5 (CRMP5) induces mitophagy, thereby regulating mitochondrion numbers in dendrites. Journal of Biological Chemistry, 289(4), 2261–2276. 10.1074/jbc.M113.490862 24324268 PMC3900971

[brb31528-bib-0005] Brot, S. , Malleval, C. , Benetollo, C. , Auger, C. , Meyronet, D. , Rogemond, V. , … Moradi‐Améli, M. (2013). Identification of a new CRMP5 isoform present in the nucleus of cancer cells and enhancing their proliferation. Experimental Cell Research, 319(5), 588–599. 10.1016/j.yexcr.2012.12.011 23298946

[brb31528-bib-0006] Dalmau, J. , & Vincent, A. (2017). Do we need to measure specific antibodies in patients with limbic encephalitis? Neurology, 88(6), 508–509. 10.1212/WNL.0000000000003592 28062718

[brb31528-bib-0007] Dericioglu, N. , Gocmen, R. , & Tan, E. (2018). Paraneoplastic striatal encephalitis and myelitis associated with anti‐CV2/CRMP‐5 antibodies in a patient with small cell lung cancer. Clinical Neurology & Neurosurgery, 170, 117 10.1016/j.clineuro.2018.05.010 29777943

[brb31528-bib-0008] Gleichman, A. J. , Panzer, J. A. , Baumann, B. H. , Dalmau, J. , & Lynch, D. R. (2014). Antigenic and mechanistic characterization of anti‐AMPA receptor encephalitis. Annals of Clinical & Translational Neurology, 1(3), 180–189. 10.1002/acn3.43 24707504 PMC3972064

[brb31528-bib-0009] Gold, M. , Pul, R. , Bach, J. P. , Stangel, M. , & Dodel, R. (2012). Pathogenic and physiological autoantibodies in the central nervous system. Immunological Reviews, 248(1), 68–86. 10.1111/j.1600-065X.2012.01128.x 22725955

[brb31528-bib-0010] Grativvol, R. S. , Cavalcante, W. C. P. , Castro, L. H. M. , Nitrini, R. , & Simabukuro, M. M. (2018). Updates in the diagnosis and treatment of paraneoplastic neurologic syndromes. Current Oncology Reports, 20(11), 92 10.1007/s11912-018-0721-y 30415318

[brb31528-bib-0011] Höftberger, R. , van Sonderen, A. , Leypoldt, F. , Houghton, D. , Geschwind, M. , Gelfand, J. , … Dalmau, J. (2015). Encephalitis and AMPA receptor antibodies: Novel findings in a case series of 22 patients. Neurology, 84(24), 2403–2412. 10.1212/WNL.0000000000001682 25979696 PMC4478035

[brb31528-bib-0012] Honnorat, J. , & Cartalat, C. S. D. (2009). Onco‐neural antibodies and tumour type determine survival and neurological symptoms in paraneoplastic neurological syndromes with Hu or CV2/CRMP5 antibodies. Journal of Neurology Neurosurgery & Psychiatry, 80(4), 412–416. 10.1136/jnnp.2007.138016 PMC2664637 18931014

[brb31528-bib-0013] Horta, E. S. , Lennon, V. A. , Lachance, D. H. , Jenkins, S. M. , Smith, C. Y. , McKeon, A. , … Pittock, S. J. (2014). Neural autoantibody clusters aid diagnosis of cancer. Clinical Cancer Research, 20(14), 3862–3869. 10.1158/1078-0432.CCR-14-0652 24833664

[brb31528-bib-0014] Josep, D. , & Rosenfeld, M. R. (2008). Paraneoplastic syndromes of the CNS. Lancet Neurology, 7(4), 327–340. 10.1016/S1474-4422(08)70060-7 18339348 PMC2367117

[brb31528-bib-0015] Lai, M. , Hughes, E. G. , Peng, X. , Zhou, L. , Gleichman, A. J. , Shu, H. , … Dalmau, J. (2009). AMPA receptor antibodies in limbic encephalitis alter synaptic receptor location. Annals of Neurology, 65(4), 424–434. 10.1002/ana.21589 19338055 PMC2677127

[brb31528-bib-0016] Laurido‐Soto, O. , Brier, M. R. , Simon, L. E. , McCullough, A. , Bucelli, R. C. , & Day, G. S. (2019). Patient characteristics and outcome associations in AMPA receptor encephalitis. Journal of Neurology, 266(2), 450–460. 10.1007/s00415-018-9153-8 30560455 PMC6367044

[brb31528-bib-0017] Melzer, N. , & Wiendl, H. (2013). Paraneoplastic and non‐paraneoplastic autoimmunity to neurons in the central nervous system. Journal of Neurology, 260(5), 1215–1233. 10.1007/s00415-012-6657-5 22983427 PMC3642360

[brb31528-bib-0018] Panzer, J. A. , & Dale, R. C. (2015). Anti‐AMPA receptor encephalitis: The family of glutamatergic autoencephalitides further expands. Neurology, 84(24), 2390–2391. 10.1212/WNL.0000000000001693 25979697

[brb31528-bib-0019] Rajesh, P. , & Bernhard, L. (2013). Insights into the oligomerization of CRMPs: Crystal structure of human collapsin response mediator protein 5. Journal of Neurochemistry, 125(6), 855–868. 10.1111/jnc.12188 23373749

[brb31528-bib-0020] Seluk, L. , Taliansky, A. , Yonath, H. , Gilburd, B. , Amital, H. , Shoenfeld, Y. , & Kivity, S. (2019). A large screen for paraneoplastic neurological autoantibodies; diagnosis and predictive values. Clinical Immunology, 199, 29–36. 10.1016/j.clim.2018.12.007 30543927

[brb31528-bib-0021] Shepherd, J. D. , & Huganir, R. L. (2007). The cell biology of synaptic plasticity: AMPA receptor trafficking. Annual Review of Cell and Developmental Biology, 23, 613–643. 10.1146/annurev.cellbio.23.090506.123516 17506699

[brb31528-bib-0022] Tomotaka, Y. , & Shoji, T. (2010). Neurological syndromes, encephalitis. Gan to Kagaku Ryoho Cancer & Chemotherapy, 37(6), 995–1005. 20567100

[brb31528-bib-0023] Tuzun, E. , & Dalmau, J. (2007). Limbic encephalitis and variants: Classification, diagnosis and treatment. Neurologist, 13(5), 261–271. 10.1097/NRL.0b013e31813e34a5 17848866

[brb31528-bib-0024] Xiaoyu, P. , Ethan, G. H. , Emilia, H. M. , Thomas, D. P. , Josep, D. , & Rita, J.‐ B.‐G. (2015). Cellular plasticity induced by anti‐α‐amino‐3‐hydroxy‐5‐methyl‐4‐isoxazolepropionic acid (AMPA) receptor encephalitis antibodies. Annals of Neurology, 77(3), 381–398. 10.1002/ana.24293 25369168 PMC4365686

[brb31528-bib-0025] Yang, S. , Qin, J. , Li, J. , Gao, Y. , Zhao, L. U. , Wu, J. , … Sun, S. (2016). Rapidly progressive neurological deterioration in anti‐AMPA receptor encephalitis with additional CRMP5 antibodies. Neurological Sciences, 37(11), 1–3. 10.1007/s10072-016-2680-0 27465029

