# Supplementary material for: Limbic encephalitis associated with AMPA receptor and CRMP5 antibodies: A case report and literature review
Source: Brain Behav. 2020 Jan 28;10(3):e01528. doi: 10.1002/brb3.1528 (PMC7066334; doi:10.1002/brb3.1528)
Supplement: Supplementary file 1 [file BRB3-10-e01528-s001.docx]

Appendix

A 26-year-old previously healthy man was admitted to the hospital due to consciousness disturbance and involuntary limb activity for 8 days. Initially, he presented with sleep dysfunction, accompanied by poor appetite, nausea, vomiting, and scalp numbness for 20 days. Two weeks prior, the patient visited the psychiatry department as an outpatient. He was diagnosed with sleep dysfunction and insomnia. Zopiclone and alprazolam tablets were prescribed to improve sleep, but the tablets had no effect. On the day of the visit, he developed severe weakness and abnormal movements in both legs. Moreover, he also presented bladder dysfunction but could still urinate by himself. One week prior to admission, he could not speak single words, recognize familiar people, consume food, or swallow by himself. The next day, he developed consciousness disturbance, involuntary limb movements and acute urinary retention. The patient presented insignificant weight loss within the previous year. He did not consume alcohol or smoke cigarettes. He was admitted to a local hospital, and complete blood counts and biochemistry tests were performed. Routine serum analyses indicated hyponatraemia (sodium concentration: 110.8 mmol/L), and an electrocardiogram (ECG) indicated atrial tachycardia (HR: 124b/min). Due to progressive aggravation of the disease, he was referred to our hospital (Dec 18, 2018). Upon admission, he presented consciousness disturbance and had difficulty following commands. A physical neurological examination revealed that he exhibited visible involuntary movement of the limbs and had high muscle tension in the limbs, corresponding hyperactive deep tendon reflexes and a positive bilateral Babinski response.

His complete blood count, biochemistry levels and blood tumour markers (alpha-fetoprotein, CA-125, CA15-3, CA19-9, and carcinoembryonic antigen) were within normal limits except for sodium levels, which resulted in hyponatraemia (sodium concentration: 116 mmol/L). CSF analysis revealed 11 leukocytes per mm3 mostly lymphocytes (87%), none red blood cells, and normal biochemistry. Paraneoplastic antibodies in serum and CSF were all negative, including anti-Hu, anti-Yo, anti-Ri, antiMa2, and anti-amphiphysin; besides, anti-CV2/CRMP5 antibodies were positive. S[imultaneously](javascript:;), neuropil antibodies (anti-NMDAR, anti-AMPAR1, anti-AMPAR2, anti-GABABR, anti-LGI1, anti-CASPR2 and anti-GAD65) in serum and CSF were also tested. Among these antibodies, AMPAR2 antibodies were detected both in the serum and CSF (Fig. 1a). When arrived at our hospital, the patient underwent a brain MRI 3 weeks after symptom onset. The brain MRI showed increased T2/fluid-attenuated inversion recovery (FLAIR)/diffusion weighted imaging (DWI) signal abnormalities involving the bilateral cerebellar hemispheres, cerebellar vermis, left hippocampus, basal ganglia region and bilateral frontal parietal cortex; however, no obvious enhancement was observed. Spectra for voxels in the cerebellar hemispheres showed a markedly reduced N-acetyl aspartate (NAA) peak and NAA/choline (Cho) ratio. A chest computed tomography (CT) scan showed a mass in the antero-superior mediastinum. Because most of the lesions were wavy and fused together and located in the anterior superior mediastinum, the mass was suspected to be lymphoma.


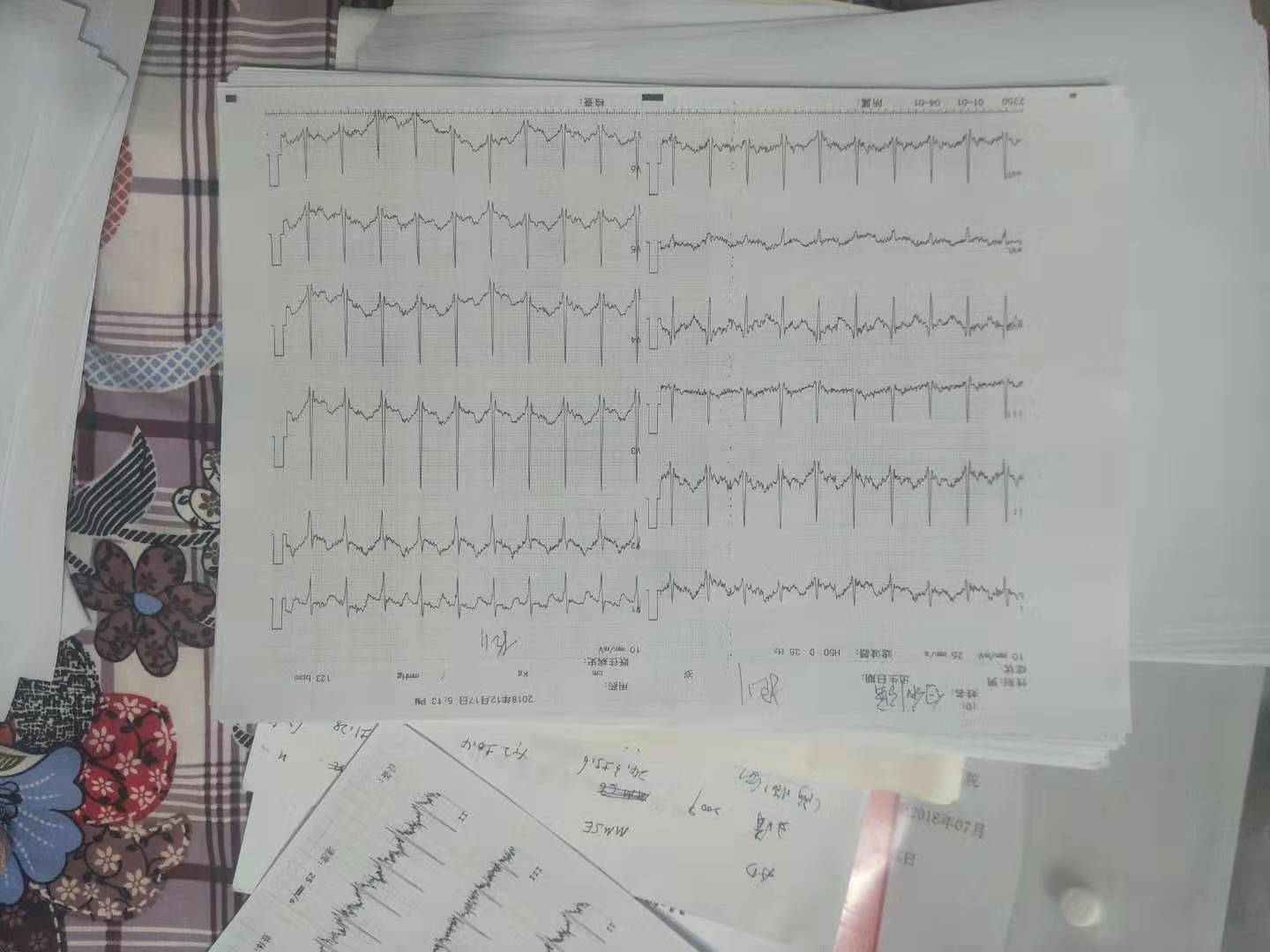
On the day of admission, both glucocorticoid (1 g/d) and intravenous immunoglobulin (IVIg) (0.4 mg/kg) therapy were administered. However, the patient's condition continued to deteriorate: his heart rate continued to increase (120~140 b/min), his blood pressure was slightly elevated, at 172/90 (130~172/90~120 mmHg), his temperature continuously increased (constantly maintained at 41°C), his hyponatraemia continued (116~122 mmol/L), and he presented progressive [consciousness disruption](file:///C:/Users/jingyan/AppData/Local/youdao/dict/Application/7.5.2.0/resultui/dict/?keyword=consciousness) and involuntary limb movement. On the second day after admission, the patient had difficulty breathing, and his blood oxygen saturation continued to decline. Tracheal intubation was performed and ventilator-assisted breathing was initiated. On the same day, the patient experienced a tonic-clonic seizure. Early on the morning of the 4th day after admission (Dec 23, 2018), the patient suffered from circulatory and respiratory failure and died.

Fig. 1 The electrocardiogram of the patient on admission


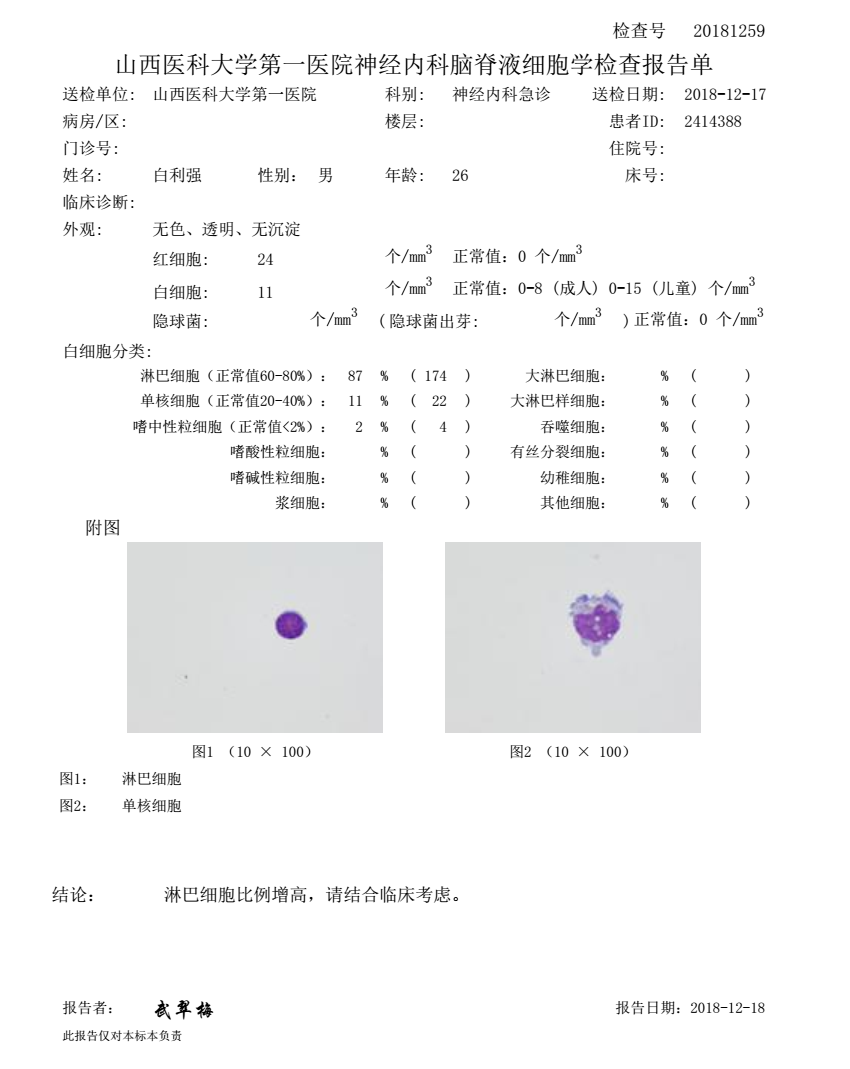


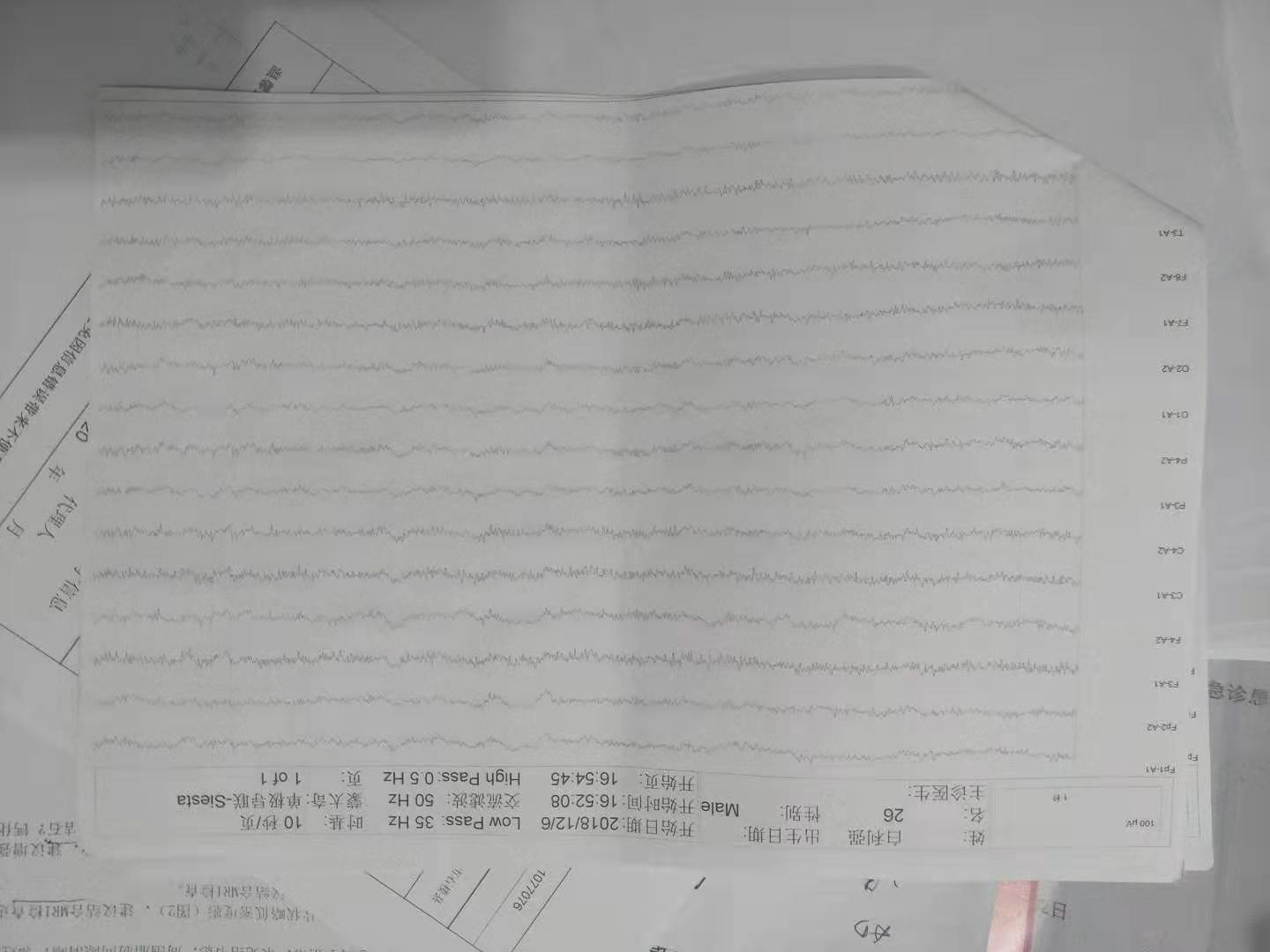
Fig. 2 The CSF report of the patient

Fig. 3 EEG of the patient during involuntary movement
